# Effects of Short-Term Soil Tillage Management on Activity and Community Structure of Denitrifiers under Double- Cropping Rice Field

**DOI:** 10.4014/jmb.2007.07012

**Published:** 2020-09-22

**Authors:** Haiming Tang, Chao Li, Kaikai Cheng, Lihong Shi, Li Wen, Xiaoping Xiao, Yilan Xu, Weiyan Li, Ke Wang

**Affiliations:** 1 Hunan Soil and Fertilizer Institute, Changsha 4025, P.R. China; 2 Hunan Biological and Electromechanical Polytechnic, Changsha 41017, P.R. China

**Keywords:** Tillage, crop residue, paddy field, soil denitrification rate, community composition

## Abstract

Soil physical and chemical characteristics, soil potential denitrification rates (PDR), community composition and *nirK*-, *nirS*- and *nosZ*-encoding denitrifiers were studied by using MiSeq sequencing, quantitative polymerase chain reaction (qPCR), and terminal restriction fragment polymorphism (T-RFLP) technologies base on short-term (5-year) tillage field experiment. The experiment included four tillage treatments: conventional tillage with crop residue incorporation (CT), rotary tillage with crop residue incorporation (RT), no-tillage with crop residue retention (NT), and rotary tillage with crop residue removed as control (RTO). The results indicated that soil organic carbon, total nitrogen and NH_4_^+^-N contents were increased with CT, RT and NT treatments. Compared with RTO treatment, the copies number of *nirK*, *nirS* and *nosZ* in paddy soil with CT, RT and NT treatments were significantly increased. The principal coordinate analysis indicated that tillage management and crop residue returning management were the most and the second important factors for the change of denitrifying bacteria community, respectively. Meanwhile, this study indicated that activity and community composition of denitrifiers with CT, RT and NT treatments were increased, compared with RTO treatment. This result showed that *nirK*, *nirS* and *nosZ*-type denitrifiers communities in crop residue applied soil had higher species diversity compared with crop residue removed soil, and denitrifying bacteria community composition were dominated by *Gammaproteobacteria*, *Deltaproteobacteria*, and *Betaproteobacteria*. Therefore, it is a beneficial practice to increase soil PDR level, abundance and community composition of nitrogen-functional soil microorganism by combined application of tillage with crop residue management.

## Introduction

Denitrification is an important microbial process in the nitrogen (N) cycling of farmland ecosystem, and it was a facultative respiratory process in which oxidized N compounds are used as alternative electron acceptors for energy production when oxygen is limited [1]. The process includes a sequence of reactions that reduce nitrate (NO_3_^−^) to dinitrogen (N_2_), passing first through nitrite (NO_2_^−^), nitric oxide (NO), and nitrous oxide (N_2_O) [2]. Previous study has been proved that denitrification is the main process of N transformation in paddy soil [3]. Bacterial nitrite reductase (NiR) was playing an important role in reducing of NO_2_^−^ to NO in denitrifying organisms [2, 4]. The functional genes *nirK* and *nirS* encode nitrate reductase. Other studies showed a close relationship between the abundance of *nirK* and *nirS* and the N_2_O emission [5, 6]. Meanwhile, the reduction of N_2_O to N_2_ were mainly catalyzed by nitrous oxide reductase (*nosZ*) [7].

It is widely accepted that soil structure, soil organic carbon (SOC), soil moisture and temperature were affected by different types of tillage and crop residue practices, which modify the soil quantity and fertility [8]. Result of previous studies, these results indicated that denitrifier activity and community structure of denitrifier were playing a vital role in regulating N emission from farmland soil [5, 6] and are in tuen regulated by different tillage practices [9]. The abundance of *nirK* and *nirS* genes was shown to increase with no-tillage (NT) practice, while the abundance of *nosZ* gene were decreased with conservation tillage (CT) practice [10]. Tatti *et al*. (2015) [11] also demonstrated that abundance of *nirK* and *nosZ* genes in paddy soil were increased with NT treatment, compared with CT treatment. Yu *et al*. (2018) [12] result found that *Magnetospirillum* and *Rhodocyclaceae* were accounted for the main component of denitrifiers in black soil. Therefore, the abundance of *nirK*, *nirS* and *nosZ* genes are often investigated to assess the N-cycle microbial activity and diversity in soil [3, 13, 14]. At the same time, soil potential denitrification rates (PDR) are usually regarded as an explaining factor controlling denitrification in the soil under different rotation cropping system, fertilizer regime, tillage and so on [7, 10, 14]. However, there is still needed on the effects of combined application of tillage with crop residue management on activity and diversity of denitrifier and nitrifier in paddy soil.

Rice (*Oryza sativa* L.) is a major grain crops around the world, and the early rice and late rice rotation system (double-cropping rice system) is also the main planting system in southern China [15], where paddy soil fertility and quality are mostly affected by combined applied tillage (rotary tillage, conventional tillage, and no-tillage) with crop residue management. The different short-term combined applied tillage with crop residue practice has had obvious effects on soil physicochemical properties such as soil pH, soil bulk density, and SOC content, which were in return affects soil microbiological characteristics and yield of rice yield [16]. Further study is needed to determine the effects of different tillage treatments on the activities and communities of denitrifiers in double-cropping rice paddy soil under continuous 5-year tillage with crop residue condition. We hypothesized that soil properties (*i.e.*, soil pH, SOC, NH_4_^+^-N, and NO_3_^-^-N contents, and availability of C and N contents) were increased by application of tillage with crop residue management which also change the activities and diversity of denitrifiers in paddy soil were also changed. Therefore, the abundances and community structure of denitrifiers in paddy soil were studied by using MiSeq sequencing, quantitative polymerase chain reaction (qPCR) and terminal restriction fragment polymorphism (T-RFLP) technologies, respectively. Hence, the objective of this study was:(1) to investigate the response of soil chemical properties response to different tillage management; and, (2) to evaluate the activity and community structure of denitrifiers (nirK, *nirS*, and *nosZ*) under different tillage management in double-cropping rice paddy soil of southern China.

## Materials and Methods

### Sites and Cropping System

The field experiment was begun in November 2015. It was located in Ningxiang County (28°07′ N, 112°18′ E) of Hunan Province, China. The annual mean precipitation, annual mean evapotranspiration, and monthly mean temperature of the experiment region, as well as soil texture and soil type, crop rotation system, soil physicochemical characteristics at tillage layer (0-20 cm) of the paddy field prior to this experiment were as described as by Tang *et al*. (2019) [16].

### Experimental Design

The experiment were included four tillage treatments: conventional tillage with crop residue incorporation (CT), rotary tillage with crop residue incorporation (RT), no-tillage with crop residue retention (NT), and rotary tillage with all crop residue removed as control (RTO). The area of the each plots were 56.0 m^2^ (7 m × 8 m), and each tillage treatments was laid out in a randomized complete block design with three replications. The total quantity of Chinese milk vetch and rice straw returned to paddy fields, C content of crop residue (Chinese milk vetch, early rice and late rice straw), depth of tillage, cultivars of early rice and late rice, fertilizer, and herbicide and irrigation management with different tillage treatments of paddy fields were as described as by Tang *et al*. (2019) [16].

### Soil Sampling

Soil samples at tillage layer (0–20 cm depth) were collected in August 2019, at the tillering stage of late rice. Soil samples were collected by randomly from six cores from each plots, and rice roots were removed from soil samples and then passed through a 2-mm mesh sieve. The fresh soil samples were placed immediately in an ice box and transported to the laboratory where they stored at -20°C until molecular analysis in the laboratory.

### Soil laboratory Analysis

**Chemical analysis.** Soil chemical properties were measured according to the methods described as by Bao (2000) [17]. Briefly, soil pH was measured with a compound electrode (PE-10, Germany) by using a soil to water ratio of 1: 2.5. Soil organic carbon (SOC) and total nitrogen (TN) concentrations were measured by using a mass spectrometer elemental analyzer (Carlo Erba 1110, CE Instruments). Soil ammonium N (NH_4_^+^-N) and nitrate N (NO_3_^-^-N) concentrations were measured by using a flow injection autoanalyzer (FLA star 5000 Analyzer, Foss, Denmark).

**Soil potential denitrification rates (PDR) and potential N_2_O emission.** The N_2_ and N_2_O concentrations of soil samples were analyzed by using a robotized sampling and analyzing system, which were analyzed according to the methods described as by Molstad *et al*. (2007) [18]. Briefly, the soil samples were measured by using a gas chromatograph (GC, Agilent 7890), and the GC conditions for analyzed N_2_O and N_2_ concentrations were equipped with electron capture detector at 330°C. Finally, the soil N_2_O and N_2_ emission rates were analyzed by using slope of linear regression between the change in concentrations of N_2_O and N_2_ versus time. Soil PDR of each tillage treatment were investigated according to the methods described as by Drury *et al*. (2007) [19]. Briefly, soil samples were placed in Erlenmeyer flask, and distilled water, glucose and nitrate were added into flask for investigate soil PDR.

**DNA extraction, qPCR, and clone library analysis of N-related functional genes.** Soil DNA extraction, qPCR, and clone library analysis of N-related functional genes were measured according to the methods described as by Throbäck *et al*. (2004) [20]. Briefly, soil DNA was extracted from 0.5 g (wet weight) samples by using the Fast DNA SPIN Kit (for soil, Bio 101, USA) according to manufacturer’s instructions. The concentration of extracted DNA were measured by determining absorbance at 260 and 280 nm on a Nano Drop 2000 UV-Vis Spectrophotometer (Thermo Scientific). The DNA quality was examined by 1% agarose gel electrophoresis.

The primers were used to determine the abundance of *nirK*, *nirS*, and *nirK*, and the thermal cycling conditions were described as by Throbäck *et al*. (2004) [20]. Purified PCR amplicons were digested with different restriction endonuclease assemblies (for *nirK*, *Sau*96I [G′GNCC] and *Hpy*CH4V [TG′CA]; for *nirS* and *nosZ*, *BccI* [CCATC(N)′_4_] and *BsaHI* [GR′CGYC]). All terminal restriction fragments (TRFs) < 50 bp and <5% of the total peak height were excluded to avoid potential noise before counting relative TRF abundances. N-related microbial community was investigated by using the number of peaks and the heights of the peaks.

qPCR reaction mixtures contained 10 μl of GoTaq qPCR Master Mix (Promega), 200 μM of each primer, 2 μl of tenfold diluted DNA template (10–20 ng), and ultraclean water to 20 μl total volume. In this study, these primers used for qPCR were the same as in a conventional PCR. Addition of bovine serum albumin and ten-fold dilution were used to decrease the inhibitory effects of coextracted substances in soil DNA samples. Plasmid DNAs of the N-related respective functional genes were extracted from soil samples DNA and serially diluted to generate a standard curve, these detailed data on standard curve, qPCR efficiency and cycling condition were as described as by Long *et al*. (2012) [21].

**Illumina MiSeq sequencing.** The soil denitrifying bacterial communities were analyzed according to the methods described as by Jung *et al*. (2011) [22]. Briefly, PCR amplification was conducted by using a Gene Amp PCR-System 9700 in a total volume of 20 μl containing dNTPs (2 μl), 5 × FastPfu Buffer (4 μl), FastPfu Polymerase (0.4 μl), primer (0.8 μl), and template DNA (10 ng). Amplicons were extracted from 2% agarose gels and purified by using the AxyPrep DNA Gel Extraction Kit (Axygen Biosciences, USA). The purified amplicons were pooled in an equimolar ratio and paired-end sequenced (2 × 250) on an Illumina MiSeq platform according to the standard protocols. The operational taxonomic units (OTU) were clustered with a 97% similarity cut off by using UPARSE (version 7.1 http://drive5.com/uparse/), and an OTU table was generated for each sample for statistical analysis. The response ratio were used to determine changes in bacterial relative abundance compared to a control with a 95% confidence interval [23]. Phylotypes were identified by using the Ribosomal Database Project (RDP) pyrosequencing pipeline (http://rdp.cme.msu.edu/). Thresholds for OTU clustering were chosen based on existing literature: 80% for *nosZ*, 82% for *nirS*, and 83% for *nirK*. Raw FASTA files were quality-filtered by using QIIME software (version 1.17) with the previous criteria, and chimeric sequences were removed by using the UCHIME algorithm, The detailed information on raw FASTA files and chimeric sequences was as described as by Jung *et al*. (2011) [22].

The diversity of *nirK*, *nirS* and *nosZ*-containing communities were calculated according to the equation:

Shannonindex=−∑(ni/N)×ln(ni/N)

where, *ni* was the relative abundance of the *i*thT-RF, *i* was the numbering of each T-RF in the T-RFLP spectrum, and *N* was the total sum of relative abundances of selected T-RF for all samples in the T-RFLP spectrum.

The pattern of similarity in soil microbial community composition between different tillage treatments was indicated by using principal coordinate analysis (PCoA).

### Statistical Analysis

The results of each investigated items were presented as average value and standard error. The statistical analysis of relative data was conducted by using the SAS 9.3 software package [24]. In addition, all investigated items with different treatments in this manuscript were compared by using one-way analysis of variance (ANOVA) following standard procedures at the *p* < 0.05 probability level. The relationship between the soil properties and the abundances of denitrifying bacterial communities were analyzed by using the Pearson correlation test.

## Results

### Soil Chemical Properties

The results showed that soil pH were increased with CT, RT and NT treatments, compared with RTO treatment. However, there were no significantly differences (*p* > 0.05) in soil pH between CT, RT, NT and RTO treatments (Table 1). SOC and TN contents with RT treatment were higher (*p* < 0.05) than that of RTO treatment. Compared with RTO treatment, soil NH_4_^+^-N content with CT, RT and NT treatments was significantly increased ( *p* < 0.05), and soil NO_3_^-^-N content with RT and NT treatments was also significantly increased (*p* <0.05), respectively.

### Soil Potential Denitrification Rates and Potential N_2_O Emission

**Fig. 1:**
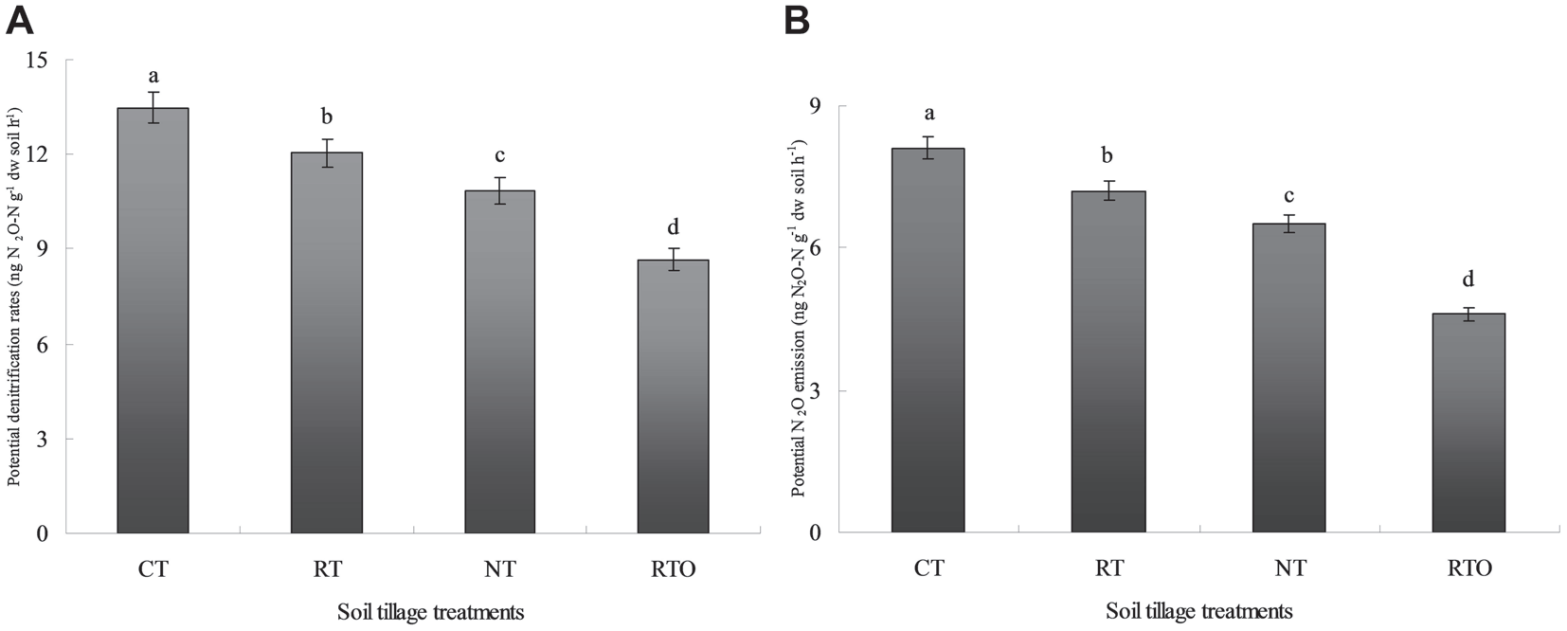
Potential denitrification rates and potential N_2_O emission in paddy soil under different tillage treatments. (**A**) Potential denitrification rates; (**B**) Potential N_2_O emission. CT: conventional tillage with crop residue incorporation; RT: rotary tillage with crop residue incorporation; NT: no-tillage with crop residue retention; RTO: rotary tillage with crop residue removed as control. Vertical bars represent the standard deviation (*n* = 3) and different lowercase letters indicated significantly differences among tillage treatments in paddy soil at *p* < 0.05. The same as below.

The results indicated that soil potential denitrification rates (PDR) were profound affected by different tillage treatments (Fig. 1A). The soil PDR with different tillage treatments were ranged from 8.65 to 13.46 ng N_2_O-N g^-1^ dw soil h^-1^, and the soil PDR with CT, RT and NT treatments were higher (*p* < 0.05) than that of RTO treatment. The PDR was the highest with CT treatment and the lowest with RTO treatment in paddy soil (13.46 and 8.65 ng N_2_O-N g^-1^ dw soil h^-1^, respectively). Additionally, compared with RTO treatment, the PDR in paddy soil were 25.43%-55.61% higher with CT, RT and NT treatments. Although the soil PDR were also increased with RT and NT treatments, the decrease of ranged from 10.48% to 19.39% with RT and NT treatments compared with CT treatment (Fig. 1A).

The potential N_2_O emission with different tillage treatments were ranged from 4.6 to 8.1 ng N_2_O-N g^-1^ dw soil h^-1^ (Fig. 1B). The potential N_2_O emission in paddy soil was the highest with CT treatment and the lowest with RTO treatment (8.1 and 4.6 ng N_2_O-N g^-1^ dw soil h^-1^, respectively). Compared with RTO treatment, the potential N_2_O emission in paddy soil with CT, RT and NT treatments was significantly increased (*p* < 0.05).

### Abundance of Denitrifiers Harboring *nirS*, *nirK* and *nosZ*

**Fig. 2:**
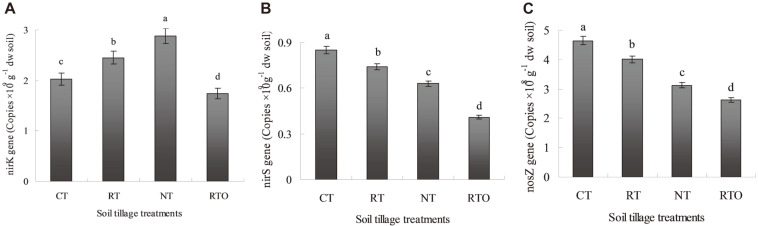
Abundance of *nirK*, *nirS* and *nosZ* genes in paddy soil under different tillage treatments. (**A**) Abundance of *nirK* gene; (**B**) abundance of *nirS* gene; (**C**) abundance of *nosZ* gene.

These results indicated that abundance of *nirK* gene with CT, RT, NT and RTO treatments were ranged from 1.74 to 2.88 copies × 10^9^ g^-1^ dw soil in paddy soil. Compared with NT and RTO treatments, the abundances of *nirK* gene with CT and RT treatments were significantly increased (*p* < 0.05) (Fig. 2A). Moreover, the abundances of *nirS* gene with CT, RT, NT, and RTO treatments were ranged from 0.41 to 0.85 copies × 10^9^ g^-1^ dw soil in paddy soil, while the abundances of *nirS* gene with CT, RT and NT treatments were significantly higher (*p* < 0.05) than that of RTO treatment in paddy soil (Fig. 2B). These results indicated also that abundances of *nosZ* gene with CT, RT, NT and RTO treatments were ranged from 2.62 to 4.65 copies × 10^8^ g^-1^ dw soil in paddy soil. Compared with RTO treatment, the abundances of *nosZ* gene with CT, RT and NT treatments were significantly increased (Fig. 2C).

The correlation between the abundance of denitrification marker genes (*nirK*, *nirS*, and *nosZ*) and soil characteristics were shown in Table 2. There was a significantly positive correlation (*p* < 0.05) between abundance of *nosZ* gene and soil NH_4_^+^-N content (*r* = 0.62). There was a significantly positive correlation (*p* < 0.05) between abundance of *nirK* gene and soil TN content (*r* = 0.54), SOC content (*r* = 0.53), and NH_4_^+^-N content (*r* = 0.65).

However, there was no significantly (*p* > 0.05) correlation between abundance of *nirS* gene and soil characteristics. Meanwhile, there was a negatively correlation (*p* < 0.05) between abundance of *nirK* gene and soil pH (*p* < 0.01; *r* = −0.83).

### Community Composition of Denitrifiers Harboring *nirK*, *nirS*, and *nosZ*

**Fig. 3:**
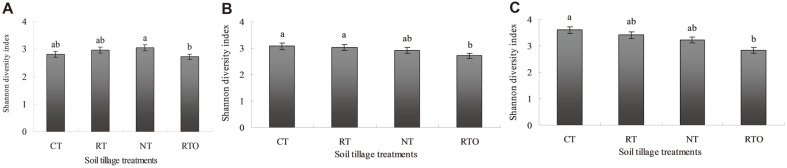
Shannon diversity index of *nirK*, *nirS*, and *nosZ* library in paddy soil under different tillage treatments. (**A**) Shannon diversity index of *nirK* gene; (**B**) Shannon diversity index of *nirS* gene; (**C**) Shannon diversity index of *nosZ* gene.

**Shannon diversity index of *nirK*, *nirS* and *nosZ*.** These results indicated that Shannon diversity index of *nirK* in paddy soil with NT treatment were significantly higher (*p* < 0.05) than that of RTO treatment. The Shannon diversity index of *nirK* in paddy soil was significantly increased (*p* < 0.05) with CT, RT and NT treatments (Fig. 3A). The Shannon diversity index of *nirS* in paddy soil with RTO treatment was significantly lower (*p* < 0.05) than that of CT and RT treatments (Fig. 3B), indicating that this diversity index in paddy soil was decreased under crop residue removed condition. Also, the Shannon diversity index of *nosZ* in paddy soil with CT treatment were significantly higher (*p* < 0.05) than that of RTO treatment (Fig. 3C), indicating that this diversity index in paddy soil was enhanced under combined application of tillage with crop residue condition.

**Fig. 4:**
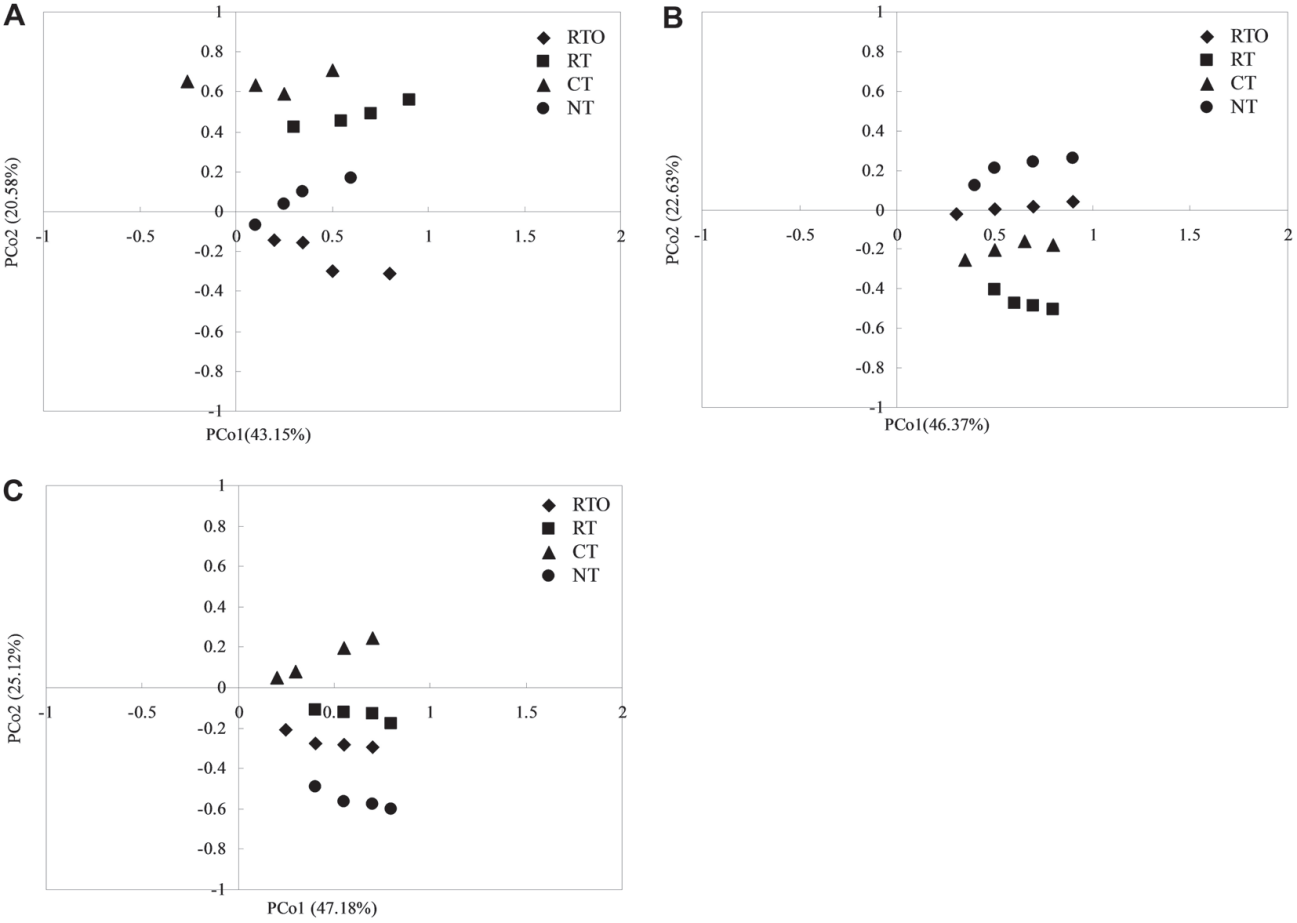
Principal coordinate analysis (PCoA) of *nirK*, *nirS*, and *nosZ* library in paddy soil under different tillage treatments. (**A**) PCoA of *nirK* gene; (**B**) PCoA of *nirS* gene; (**C**) PCoA of *nosZ* gene.

**PCoA of *nirK*, *nirS*, and *nosZ*.** In the present study, the PCoA analysis indicated that the changes of the first principal coordinates (PCoA1) of *nirK*, *nirS*, and *nosZ* were explained as 43.15%, 46.37%, and 47.18%, respectively, demonstrating that different tillage treatments were the most important factor affecting the community of denitrifying bacteria in paddy soil (Fig. 4). Meanwhile, the change of second principal coordinates (PCoA2) of *nirK*, *nirS*, and *nosZ* were explained as 20.58%, 22.63%, and 25.12%, respectively, indicating that different crop residue returning management was the second most important factor affecting the community of denitrifying bacteria in paddy soil (Fig. 4).

**Fig. 5:**
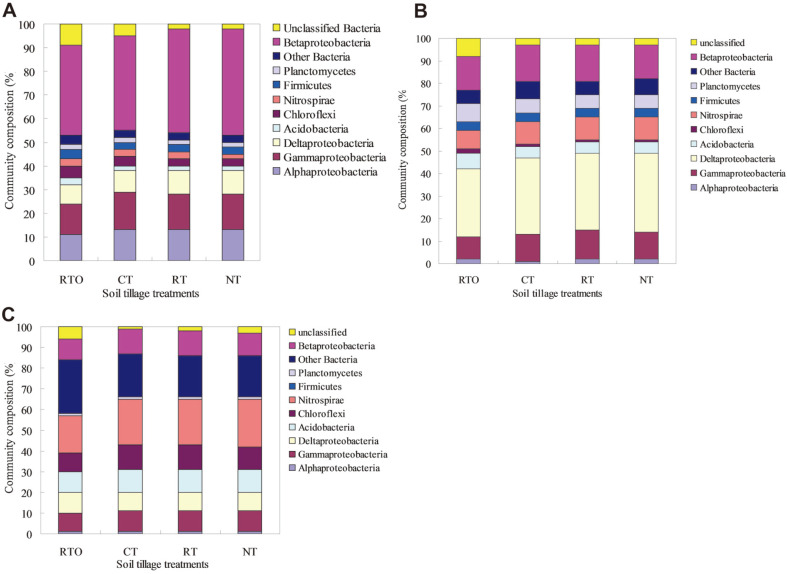
Relative abundance of the *nirK*-, *nirS*-, and *nosZ*-type denitrifying bacteria at the genus level in paddy soil under different tillage treatments. (**A**) Relative abundance of *nirK*-denitrifying bacteria; (**B**) relative abundance of *nirS*-denitrifying bacteria; (**C**) relative abundance of *nosZ*-denitrifying bacteria.

**Taxonomic distribution of *nirK*, *nirS*, and *nosZ*.** These results indicated that *nirK*-denitrifying bacteria at the genus level in paddy soil were mainly included *Alphaproteobacteria*, *Gammaproteobacteria*, *Deltaproteobacteria* and *Betaproteobacteria*. The relative abundance of *Betaproteobacteria* were occupied most of the composition in paddy soil with CT, RT, NT, and RTO treatments, and the relative abundance of *Betaproteobacteria* in paddy soil with CT, RT, and NT treatments was higher (*p* < 0.05) than that of RTO treatment. However, the unclassified bacteria with RTO treatment were higher (*p* < 0.05) than that of CT, RT and NT treatments (Fig. 5A).

The *nirS*-denitrifying bacteria at the genus level in paddy soil were mainly included *Deltaproteobacteria* with CT, RT, NT, and RTO treatments. Compared with RTO treatment, the relative abundance of *Gammaproteobacteria* and *Betaproteobacteria* in paddy soil with CT, RT, and NT treatments were significantly increased (*p* < 0.05). However, the relative abundance of Planctomycetes in paddy soil with RTO treatment was significantly higher (*p* < 0.05) than that of CT, RT, and NT treatments (Fig. 5B).

The *nosZ*-denitrifying bacteria at the genus level in paddy soil were mainly included *Nitrospirae* with CT, RT, NT, and RTO treatments. Compared with RTO treatment, the relative abundances of *Chloroflexi* and *Betaproteobacteria* in paddy soil with CT, RT, and NT treatments were significantly increased (*p* < 0.05). However, the relative abundance of the other *bacteria* and unclassified *bacteria* in paddy soil with RTO treatment were significantly higher (*p* < 0.05) than that of CT, RT, and NT treatments (Fig. 5C).

## Discussion

In the previous study, it was found that community composition and diversity of soil denitrifying bacteria were changed by different tillage treatments in which soil pH was played an important role in regulating the denitrifying microbial community and diversity in arable soil [11]. In this study, the results showed that soil pH with RTO treatment was lower, but there was no apparent affects on N cycle soil denitrifiers communities (nirK, *nirS*, and *nosZ*-type). Soil pH was the main driving factors of N-functional microbial community and diversity in soil [25], and this study revealed that there was obvious effect on soil pH between CT, RT, and NT treatments (Table 1). Compared with RTO treatment, soil NO_3_^-^-N content and soil PDR were increased with NT treatment, and the soil denitrifying microbial community and diversity were obviously affected with NT treatment. Meanwhile, the soil available ammonium, inorganic N, organic N, SOC contents, and soil denitrification activities were obviously affected with CT and RT treatment. Therefore, the combined application of tillage with crop residue management can manipulate the N-functional microbial population and then affect the properties of double-cropping paddy soil.

In the present study, there was significantly difference in soil PDR among CT, RT, and NT treatments, and the numbers of *nirS*, *nirK*, and *nosZ* genes copies with CT, RT, and NT treatments were increased compared to RTO treatment, that is, the number of soil denitrification gene copies had a significantly overall affected under combined application of tillage with crop residue condition. These findings were similar with those of Chen *et al*.[3] in which the numbers of *nirS*, *nirK*, and *nosZ* genes copies in paddy soil with crop residue management were increased compared to without fertilizer input management base on the long-term field experiment condition. It was benefitial for soil denitrifiers multiplcation with CT and RT treatments, which was related to higher bioavailability of C under these soil environment condition [26]. On the other hand, there had higher soil NH_4_^+^-N and SOC contents were higher under CT and RT conditions (Table 1), which were positively correlated with the number of *nosZ* and *nirK* genes copies in paddy soil (Table 2). These findngs were similar to those of Kandeler *et al*.[27] which showed that, SOC content was positively correlated with the number of soil denitrification gene copies (narG, *nirK*, and *nosZ*). In this study, the results indicated that communities of *nirK* and *nirS* were responded differently to tillage treatments, although both the numbers of *nosZ* and *nirK* genes copies with CT, RT and NT treatments were increased compared with RTO treatment. The numbers of *nirS* gene copies with CT and RT treatments were higher than that of NT treatment, indicated that soil denitrifiers of *nirS* growth and development maybe sensitive to combined application of tillage with crop residue management compared with no-tillage and crop residue management. In contrast, the abundance of soil denitrifiers *nirK* gene copies with CT and RT treatments were decreased compared with NT treatment. The main reason for this phenomenon in this study was maybe that the number of soil denitrifiers *nirS* gene copies depended on tillage management, crop residue and root exudates, whereas the soil denitrifiers *nirK* gene copies may have relied on tillage management, crop residue and root exudates to a lesser degree.

In the present study, we found that the abundances of soil denitrifiers *nirK* gene copies were more closely related to soil physicochemical characteristics than that of the other soil denitrifiers genes (nosZ and *nirS*). And the abundance of soil denitrifiers *nirK* gene copies were more than three times higher than that abundance of soil denitrifiers *nirS* gene copies with CT, RT, NT, and RTO treatments (Fig. 2). Meanwhile, this result indicated that one copy of *nirS* were found in bacterial genomes, whereas three copies of *nirK* were found in some species [28]. And the abundance of denitrifiers *nirK* were higher than that of abundance of denitrifiers *nirS* in paddy soil for that all nitrite-reducing bacteria have either *nirK* or *nirS*. These founds were similar to Yoshida *et al*. [13] result, which also had found abundance of soil denitrifiers *nirK* were higher than that of abundance of soil denitrifiers *nirS*. However, Kandeler *et al*. [27] study showed a reversed result that abundant of denitrifiers *nirS* were higher than that of abundant of denitrifiers *nirK* in glacier soil. In the present study, there had higher abundant of denitrifiers *nirK* in paddy soil, but there is still little information about this phenomenon could be related to different fertilizer regime, irrigation pattern and other paddy field management.

In the previous studies, these results demonstrated that community composition and diversity of denitrifier were playing a vital role in regulating denitrification rate and N_2_O emission in soil [5, 6]. Therefore, the closely related to denitrifying genes (nirK, *nirS*, and *nosZ*) in the soil denitrifying microbial community were studied [13]. In the present study, the results showed that soil PDR were significantly changed under combined application of tillage with crop residue condition compared with no-tillage with crop residue management, although the *nirS* community were moderate changed with NT treatment. Wang *et al*. [10] also found that abundance of soil denitrifiers *nirK* and *nirS* genes with no-tillage treatment were lower than that of ploughing tillage treatment, while abundance of soil denitrifiers *nosZ* gene with no-tillage treatment were higher than that of ploughing tillage treatment, which was consistent with our finding in this study. Meanwhile, our results showed that abundance of denitrifiers *nirK* gene were significantly higher than that of abundance of denitrifiers *nirS* gene in paddy soil (Fig. 2), which was similar to previous results [3, 13], the reason maybe that attribute to the same type of soil and climate in this experimental region. In the present study, it was significantly to attention that number of soil denitrifiers genes copies (nirK, *nirS*, and *nosZ*) were changed under different tillage management condition [11]. For example, there had multiple number of soil denitrifiers *nirK* gene copies compared with number of soil denitrifiers *nirS* gene copies (sometimes were presented in multiple copies) [28]. Therefore, our results provide guidance for researchers to separate abundance of the special soil denitrifiers gene-carrying microorganisms, this maybe explain the difference between number of soil denitrifiers gene copies and soil potential denitrification rates.

In the present study, a remarkable discovery was that community composition and diversity of soil denitrifiers (nirK, *nirS*, and *nosZ*) were responded difference to the tillage treatments. The community composition of soil denitrifiers *nirK* and *nirS* were clearly changed between tillage treatments, and the community composition and diversity of *nosZ* were also significantly changed. That is, this results indicated that the community composition of denitrifying bacteria in double-cropping paddy soil were *nirK* and *nirS* under different tillage management condition. As a result, the higher community composition and diversity of *nirK*, *nirS*, and *nosZ* with tillage and crop residue management consistent with our hypothesis that structure of soil denitrifier communities were changed under combined application of tillage with crop residue condition. Some studies had conducted to compare with community composition and diversity of *nirK*, *nirS*, and *nosZ* with different tillage management in agricultural system. Wang *et al*. [10] indicated that denitrifying community composition of *nirK* and *nirS* were influenced by long-term plough tillage management. However, there is still need to further study on the change of *nirS* and *nosZ* community responding to environmental factor. Although the denitrifying community composition of *nirS* and *nosZ* in soil were needed to further magnify for that primer specificity problem [29]. In the present study, these primers used for qPCR were same as the conventional PCR were conducted following previous study [20]. Our results suggested that community composition of denitrifiers *nirK* and *nirS* in paddy soil were more sensitive to tillage treatments than that of community composition of soil denitrifiers *nosZ*, which was consistent with a previous research [30], who found that napA and narG genes had different responses to tillage management. Therefore, these results indicated that community composition and diversity related to soil denitrification were respond difference to tillage and crop residue management changes.

In the present study, we demonstrated that soil bacterial community composition and diversity of denitrifiers were significantly changed under combined application of tillage with crop residue condition. For instance, the principal coordinate analysis revealed that soil bacterial community composition of denitrifiers were also changed under different tillage treatments condition (Fig. 4). These findings are consistent with previous research showing that soil ammonia-oxidizing bacterial communities were changed by application of different tillage treatment at the same region [12], Our findings demonstrated that tillage management was the dominant factor in regulating soil bacterial community composition of denitrifiers. Other results have also found that community composition of soil denitrifiers responds differently according to tillage management [11], and these differences have been ascribed to change in soil pH, SOC content or other soil properties. Meanwhile, in the present study, the Pearson correlation coefficients analysis indicated that there had significantly correlation between bacterial community composition of soil denitrifiers and soil properties (Table 2), implying that bacterial community composition of soil denitrifiers in this region was obviously affected by the change of soil parameters, which also indicated that different tillage practices were also another significant factor changing the soil denitrifying bacteria community.

In this study, we showed that community composition of soil denitrifiers *nirK*, *nirS*, and *nosZ* were significantly changed by combined application of tillage with crop residue management, a significantly increased in the diversity was also observed (Figs. 3-5), and denitrifying bacteria community composition were dominated by *Gammaproteobacteria*, *Deltaproteobacteria* and *Betaproteobacteria*. The predominance of *nirK*, *nirS*, and *nosZ* diverse denitrifying bacteria was also consistent with the results of a previous analysis of paddy soil [30]. In the present study, the dominant relative abundance of *nirK* and *nosZ* obtained were related to *Gammaproteobacteria*. Contrary, Chen *et al*. [3] result indicated that the dominant relative abundance of *nosZ* obtained were related to *Alphaproteobacteria* and *Betaproteobacteria* in paddy soil under long-term fertilizer condition. However, Masuda *et al*. [31] result found that most *nirS* OTUs were attached to the *Betaproteobacteria* in paddy soil. It was possible that *nirS*-carrying *Betaproteobacteria* were partial denitrifying bacterial, and lacking enzymes downstream of NiR, only two-thirds of NiR or nitric oxide reductase harbor N_2_OR [2]. Alternatively, we used primers that are highly specific for *Deltaproteobacteria* *nosZ* used primers were investigated in the present study. Therefore, this study demonstrated that soil microbial communities driving nitrite or reducing of nitric oxide were difference from each other, and the major drivers of nitrite or nitric oxide reduction were *Deltaproteobacteria*, *Gammaproteobacteria* and *Betaproteobacteria*, respectively. In the previous study, the result had proved that soil denitrifiers were related to environmental factor change and the different denitrification genotypes were also difference in response to the environmental factors [3]. Soil microbial communities were altered by combined application of tillage with crop residue management for that soil properties were also changed [32]. In the present study, the Pearson’s correlations results demonstrated that community composition of soil denitrifiers was significantly affected by soil pH, NH_4_^+^-N, total N and SOC contents (Table 2). Soil NH_4_^+^-N content was the vital factor influencing on the community composition of denitrifiers and for that it was also the main substrate for denitrification. Previous study, it had proved that community composition and diversity of soil denitrifiers in selecting special functional groups were also mainly affected by soil pH [3]. Šimek and Cooper (2002) [33] result found that available organic carbon was utilized by soil microorganisms mainly influenced by soil pH, thus community structure of soil denitrifiers was also altered. Therefore, the effects of combined application of tillage with crop residue management on change in chemical characteristics (Table 1) and microbial community composition in paddy soil (Figs. 3-5) is still needs to further study. These special functional soil microbial community structures and abundances played vital roles in the cycling process of soil nitrogen. However, the relationship between tillage, crop residue management and soil nutrient cycling process were remained unclear, and the major factors affecting the structure and abundance of *nirK*, *nirS*, and *nosZ* were also needed further study to understanding the role of nitrite reducers in soil systems.

## Figures and Tables

**Table 1:** Soil pH and nutrient contents with different tillage treatments at tillering stage of late rice.

Treatments	CT	RT	NT	RTO
pH	5.82±0.17a	5.84±0.16a	5.81±0.16a	5.78±0.15a
TN (g kg^-1^)	2.17±0.06ab	2.20±0.06a	2.12±0.05ab	2.08±0.05b
SOC (g kg^-1^)	22.88±0.66a	22.45±0.64a	21.14±0.61ab	20.35±0.58b
NH_4_^+^-N (mg kg^-1^)	0.14±0.01c	0.16±0.01b	0.19±0.01a	0.11±0.01d
NO_3_^-^-N (mg kg^-1^)	0.12±0.01c	0.14±0.01b	0.16±0.01a	0.11±0.01c

CT: conventional tillage with crop residue incorporation; RT: rotary tillage with crop residue incorporation; NT: no-tillage with crop residue retention; RTO: rotary tillage with crop residue removed as control. TN: total nitrogen; SOC: soil organic carbon. Different lowercase letters in the same line were indicated significantly difference at *p* < 0.05. The same as below.

**Table 2:** Pearson’s correlations (*r*) between abundance of denitrification marker genes (*nirK*, *nirS*, and *nosZ*) and soil characteristics (*n* = 6).

Items	q*nirK*	q*nirS*	q*nosZ*
pH	-0.83^**^	-0.35	-0.41
TN	0.54^*^	-0.17	0.45
SOC	0.53^*^	-0.14	0.48
NH_4_^+^-N	0.65^*^	0.10	0.62^*^
NO_3_^-^-N	-0.22	0.24	-0.30

^*^Significantly different at *p* < 0.05. ^**^Significantly different at *p* < 0.01.
